# Progressive Crossed-Apraxia of Speech as a First Manifestation of a Probable Corticobasal Degeneration

**DOI:** 10.3233/BEN-2012-110219

**Published:** 2012-08-09

**Authors:** Frédéric Assal, Marina Laganaro, Corinne Dubois Remund, Claire Ragno Paquier

**Affiliations:** ^1^NeurologyDepartment of Clinical NeurosciencesHUG and Faculty of MedicineGenevaSwitzerland; ^2^FAPSEUniversity of Geneva40 bd du Pont-d'ArveGeneva 4Switzerland; ^3^Geriatric PsychiatryDepartment of PsychiatryHUGPetit-Bel-Air 2Chêne-BourgSwitzerland

**Keywords:** Crossed-apraxia of speech, corticobasal degeneration, peripheral agraphia

## Abstract

We present the longitudinal neurolinguistic, neuropsychological and neurologic follow-up of a 64 y.o. right-handed woman, who developed progressive apraxia of speech (PAOS), followed by peripheral agraphia then a left corticobasal syndrome (CBS). Neuroimaging (CT, MRI and FDG-PET) unequivocally showed progressive right hemispheric atrophy and hypometabolism. This particular evolution first confirms that PAOS is a phenotype of probable corticobasal degeneration (CBD). More importantly, this case underpins the neural organisation of motor planning processing in relation with speech, as well as graphic and limb praxis impairments, and constitutes a rare example of crossed-PAOS.

Apraxia of speech (AOS) is a complex motor speech disorder which has been attributed to impaired planning/programming of gestural scores for articulation [1, 2]. Progressive AOS (PAOS) has been increasingly described in the last ten years in isolation or combined with progressive non fluent aphasia (PNFA), dysarthria or orofacial apraxia, and is now considered as a marker of corticobasal degeneration (CBD) and progressive supranuclear palsy (PSP) neuropathology [3].

A 64 yo right-handed housewife with thirteen years of education, presented with speech difficulties characterized by effortful and dysprosodic speech, substitutions, additions, omissions or inversions of sounds with spontaneous auto-corrections, impaired repetition with polysyllabic words and phonetically complex sounds such as consonant clusters that gradually evolved to full AOS (see table), that were later accompanied by peripheral agraphia (preserved oral spelling and letter additions, inversions, substitutions and omissions as well as letter-stroke reduplications in handwriting, see Fig. 1) then a corticobasal syndrome (CBS). Neuropsychological and extensive neurological examination at the age of 72 years old indicated an unmistakable CBS of the left upper limb characterized by ideomotor apraxia, severe rigidity, bradykinesia, postural tremor, myoclonus, left-sided extinction on bilateral stimulation and alien hand sign (hand not recognized as her own hand). There was also a parkinsonian gait with left hand levitation. Structural and functional neuroimaging disclosed atrophy and/or hypometabolism in the right hemisphere (see Fig. 2).

This longitudinal follow-up of almost 8 years of a PAOS followed by peripheral agraphia then a left-sided corticobasal syndrome (CBS) constitutes, to our knowledge, a rare case of crossed-PAOS. We first confirm that PAOS can be isolated for several years, then followed by a CBS and therefore constitute a marker of CBD/PSP spectrum [2]. PAOS associated with Alzheimer's disease neuropathology has been reported

ISSN 0953-4180/12/$27.50 © 2012 – IOS Press and the authors. All rights reserved

Table 1

| Neuropsychological findings (age of JE) | (64–65 yo) | (67 yo) | (69 yo) | (71 yo) | (72 yo) |
|---|---|---|---|---|---|
| MMSE | NE | 27/30 | 27/30 | NE | NE |
| Episodic memory deficits | N | N | N | + | NE |
| Executive dysfunctions | N | N | + | ++ | NE |
| Left ideomotor apraxia | − | + | ++ | ++ | + + + |
| Deficits in visuospatial skills/ Complex visual abilities | − | − | + | + | + |
| Neurological findings | | | | | |
| Rigidity (R), bradykinesia (B), tremor (T) | − | − | − | + left upper limb R and B | ++ left upper limb R and B; left arm postural T |
| Dystonia | − | − | − | − | +, left upper limb |
| Myoclonus | − | − | − | − | +, left upper limb |
| Parkinsonian gait | − | − | − | + | ++, left arm levitation |
| Parietal sensory deficits | − | − | − | NE | Left tactile extinction and astereognosia |
| Alien hand sign | NE | NE | NE | NE | + |
| Neurolinguistic findings | | | | | |
| Spontaneous speech | subjective complaints only, rare phonemic errors (+) | slightly dysprosodic, phonemic (++) and phonetic (+) errors | dysfluent, effortful (+), dysprosodic, phonemic (+ + +) and phonetic (++) errors, iterations of syllables | dysfluent, effortful (++), phonemic (+ + +) and phonetic (+ + +) errors with stuttering at the beginning of words | dysfluent (+ + +), effortful (+ + +), almost unintelligible, dysprosodic (++) |
| Confrontation naming score | 100% | 90% | 95% | 90% | impossible |
| Phonetic and phonemic errors in different modalities | 8% | 10% | 25% | 40% | 95% |
| Repetition score: | | | | | |
| − words 1-2-3 syllables | 100% | 90% | 80% | NE | 95% |
| − words 4 syllables | 90% | 80% | 60% | | 100% |
| − words 5 syllables | 50% | 30% | 10% | | |
| − words 6 syllables | 20% | 25% | | | |
| Hand-writing | N | N | N | N | N |
| Writing (peripheral errors) | + | ++ | ++ | + + + | + + + |
| Spelling aloud | N | N | N | N | N |
| Orofacial apraxia | + | + | ++ | ++ | + + + |

Longitudinal progression of neuropsychological, neurological and neurolinguistic findings. Neuropsychological and neurolinguistic tests were compared to published norms (see references). Slight impairment (+), moderate impairment (++), severe impairment (+++); absent (-). Left (L), right (R). Normal (N). Not evaluated (NE).

For neuropsychological testing, we used the French version of the MMSE [4]. Verbal episodic memory was assessed with the French version of the Free and Cued Selective Reminding Test [5], complex visual abilities with the Poppelreuter overlapping figures, adapted from Luria [6], Kanizsa's triangles [7] and the Barbizet complex image [8], visuoconstructive abilities with the figures from the CERAD [9] and the French version of the MMSE [4], executive functions with the Trail Making Test A and B [10], the Luria's graphic sequence test [6] and the top/top from the FAB [11].

For neurolinguistic testing, confrontation naming was assessed with the Bachy 90-item battery [12], auditory and written language comprehension, and writing with specific subtests of the Montreal-Toulouse battery [13], including written descriptions of the Bank Robbery Picture, and the Cookie Theft Picture [14], and oral spelling with the French version of the WAIS III [15].

in only one single case report [16], and spared episodic memory together with the absence of hippocampal atrophy and/or temporo-parietal/posterior cingulate hypometabolism do not support such a diagnosis in our case.

In right-handers, AOS involves the left inferior frontal, the anterior insula, fronto-subcortical white matter, or basal ganglia supporting a left hemisphere dominance of motor speech planning – see ref [17] for review and [18] – except in two cases with stroke [19, 20], one of which was poorly lateralized [20]. In a recent PET study with 5 cases of PAOS or combined PAOS and PNFA, hypometabolism was localized in the left superior, medial and inferior frontal gyrus, and SMA [21], although the same group showed bilateral atrophy using VBM in 6 cases with AOS. Four of them presented with ideomotor apraxia but the authors did not detail neither the side of it, the handedness of the

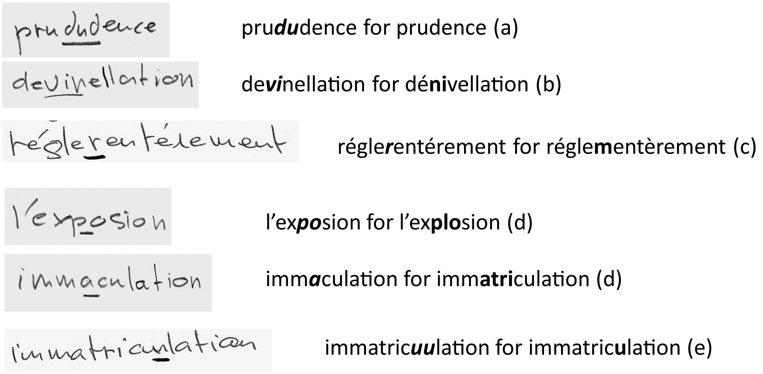

Fig. 1. Handwriting showing letter additions (a), inversions (b), substitutions (c) and omissions (d) as well as letter-stroke reduplications (e) – together with preserved oral spelling – demonstrating peripheral agraphia.

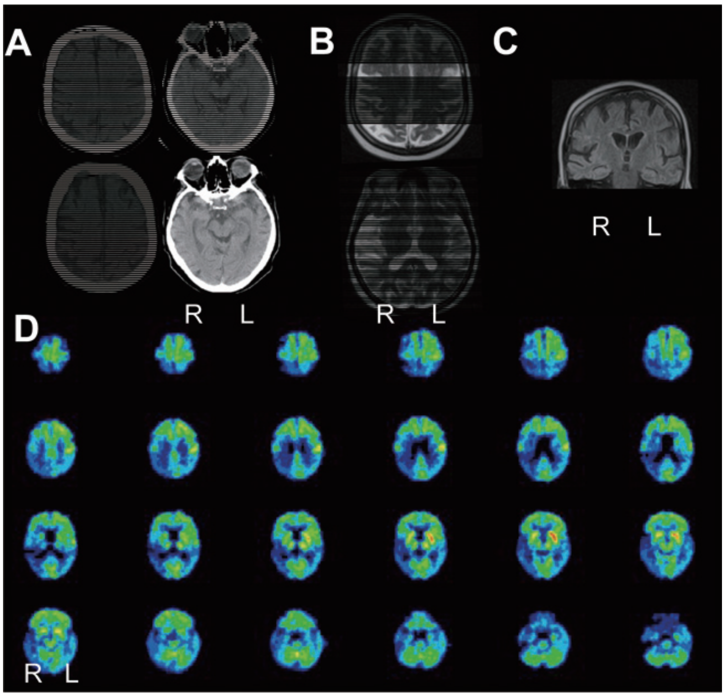

Fig. 2. [A] Right parietal atrophy on the first CT-scan when she was 67 y.o. (top) progressing to the right frontal regions including prefrontal cortex, motor cortex and frontal poles on both sides after 5 years (bottom), without any significant hippocampal atrophy. [B] T2-MRI at the age of 66 showing right perisylvian/right insula atrophy. [C] Coronal plane of FLAIR-MRI showing absence of significant hippocampal atrophy [D] Hypometabolism in the right hemisphere on 18F-FDG PET-scan (performed when she was 72 y.o.), extending from the parietal to frontal, temporal and occipital lobes, as well as right thalamus, putamen, pallidum and caudate nucleus; those changes are particularly severe posterior to the right sensorimotor cortex in the parietal lobe including both superior and inferior parietal lobules, right posterior insula and the adjacent right parietal opercule. All views are axial slices except [C]. R = right; L = left. (Colours are visible in the online version of the article; http://dx.doi.org/10.3233/BEN-2012-110219)

patients, nor any peripheral agraphia [22]. In our case, (i) speech difficulties unequivocally worsened together with progressive atrophy and hypometabolism in the right hemisphere (including frontal and insular cortices as well as pallidum); (ii) progression of AOS clearly paralleled progression of peripheral agraphia – the latter being linked to left (dominant) hemisphere involvement in right-handed patients with progressive peripheral agraphia [23,24]; (iii) there was never any superimposed atrophy or cortical/subcortical hypometabolism in the left hemisphere, even at late stages; (iv) the *left* CBS reinforces the right hemisphere involvement in our case, according to 18-FDG-PET findings where hypometabolic loci were contralateral to the upper limb apraxia [25], unlike in other cases with progressive peripheral agraphia who developed right-sided CBS and disclosed left (dominant) hemisphere hypoperfusion [23,24].

Presence of peripheral agraphia (PA) strengthens links between speech and writing progressive impairment. Luchelli and Pagano already suggested that peripheral writing deficits do not have a linguistic basis in PAOS but are also due to the involvement of motor-speech processes [26]. They based this on the observation that defective articulatory rehearsal had a detrimental effect on phonological sequencing in word production, both in the oral and written modality. Conversely, Luzzi and Picirilli reported on a patient with progressive dysgraphia, who consecutively developed AOS with no positive signs of aphasia [27]. Due to the great asynchrony between the onsets of AOS and PA, these authors suggested a progressive degeneration of two functionally separate subsystems. Heilmann proposed that the presence of asymmetric apraxic agraphia, or the inability to form letters, might constitute the beginning of CBD [28]. One of his cases that presented with asymmetrical limb apraxia demonstrated a preserved ability to spell words that he could not write, providing evidence that his writing disorder was not induced by a language deficit, but rather a deficit in programming the movements needed to write letters. From our findings, we can at least conclude that impairments of motor programming of speech co-occur with impairment of motor planning for writing.

In summary, the presentation of AOS in a right-handed female, followed by peripheral agraphia and a left CBS, associated with right-hemisphere anomalies, constitutes a unique case of *crossed*-PAOS in the course of a probable CBD/PSP pathology. It also underpins the neural organisation of motor planning processing in relation with speech, as well as graphic and limb praxis impairments.
